# Detection and molecular characterization of *Trypanosoma theileri* in cattle from the municipality of Vassouras, Rio de Janeiro, Brazil

**DOI:** 10.1590/S1984-29612026008

**Published:** 2026-05-15

**Authors:** Jônathan David Ribas Chagas, Matheus Dias Cordeiro, Aline Maria Andrade da Silva, Ana Paula Martinez de Abreu, Renata Fernandes Ferreira de Moraes, Erica Cristina Rocha Roier, Maristela Peckle, Bruna de Azevedo Baêta

**Affiliations:** 1 Universidade Federal Rural do Rio de Janeiro - UFRRJ, Departamento de Parasitologia Animal, Seropédica, RJ, Brasil; 2 Universidade de Vassouras - Univassouras, Vassouras, RJ, Brasil

**Keywords:** Molecular biology, diagnosis, blood smear, Trypanosoma theileri, Woo technique, Biologia molecular, diagnóstico, esfregaço sanguíneo, Trypanosoma theileri, técnica de Woo

## Abstract

This study aimed to detect, using parasitological and molecular methods, and to genetically characterize *Trypanosoma* spp. in cattle from the municipality of Vassouras, Rio de Janeiro, Brazil. A total of 226 blood samples were collected from dairy cattle across eight farms, and DNA was extracted for PCR amplification of the 18S rRNA gene of *Trypanosoma* spp. In parasitological examinations, including blood smears and the Woo’s technique, no trypomastigote forms of *Trypanosoma* spp. were observed. Molecular detection via 18S rRNA identified the parasite in 0.88% (2/226) of the samples. The resulting amplicons were sequenced, aligned, and compared with GenBank sequences for phylogenetic analysis. The analyses confirmed 100% identity with *T. theileri*, prompting further molecular characterization using the 24Sα rRNA (257 pb) and *CatL* (295 pb) genes. Phylogenetic analysis of *T. theileri* indicated circulation of the TthI lineage, encompassing genotype IB, in the municipality of Vassouras, Rio de Janeiro. These findings expand knowledge on the geographic distribution of *T. theileri* in Brazil and underscore the importance of monitoring cattle herds, considering the economic and health significance of trypanosomatids.

## Resumo

Este estudo teve como objetivo detectar, por meio de métodos parasitológicos e moleculares, e caracterizar geneticamente *Trypanosoma* spp. em bovinos do município de Vassouras, Rio de Janeiro, Brasil. Um total de 226 amostras de sangue foi coletado de bovinos leiteiros em oito propriedades, e o DNA foi extraído para amplificação por PCR do gene 18S rRNA de *Trypanosoma* spp. Nos exames parasitológicos, incluindo esfregaços sanguíneos e a técnica de Woo, não foram observadas formas tripomastigotas de *Trypanosoma* spp. A detecção molecular por meio do gene 18S rRNA identificou o parasito em 0,88% (2/226) das amostras. Os amplicons obtidos foram sequenciados, alinhados e comparados com sequências do GenBank para análise filogenética. As análises confirmaram 100% de identidade com *Trypanosoma theileri*, motivando uma caracterização molecular adicional utilizando os genes 24Sα rRNA (257 pb) e *CatL* (295 pb). A análise filogenética de *T. theileri* indicou a circulação da linhagem TthI, incluindo o genótipo IB, no município de Vassouras, Rio de Janeiro. Esses achados ampliam o conhecimento sobre a distribuição geográfica de *T. theileri* no Brasil e ressaltam a importância do monitoramento de rebanhos bovinos, considerando a relevância econômica e sanitária dos tripanossomatídeos.

## Introduction

*Trypanosoma* (Kinetoplastida, Trypanosomatidae) are unicellular, flagellated parasitic protozoa. Within this family, hematophagous arthropods act as biological and/or mechanical vectors of different *Trypanosoma* species, infecting a wide range of vertebrate hosts (Borges et al., 2021).

*Trypanosoma (Megatrypanum) theileri* is considered the largest trypanosome found in the blood of mammals, affecting cattle, buffaloes, and cervids. Transmission occurs mainly via tabanid flies, although mosquitoes of the genus *Aedes* (Culicidae) have also been reported as potential biological vectors (Brotánková et al., 2022). In Brazil, due to the absence of the tsetse fly, transmission *T. vivax* occurs mechanically through hematophagous insects that transfer the parasite between animals without development in the invertebrate host, where it remains in the trypomastigote form (Bastos et al., 2017). Additionally, iatrogenic transmission may occur through contaminated sharp instruments, such as needles, syringes, and palpation gloves, shared among animals (Leal et al., 2025). *Trypanosoma evansi*, the causative agent of surra, is distributed across Asia, Africa, and South America, and is transmitted mechanically by biting flies, including Tabanidae and *Stomoxys* species (Brun et al., 1998).

The occurrence of *T. theileri* has been reported on all continents, with higher prevalence in tropical and neotropical regions (Rodrigues et al., 2006). Diagnostic methods for this protozoan can be classified as direct, when morphological identification of the parasite is possible through techniques such as stained blood smear microscopy, the Woo technique, or hemoculture, and molecular, when detection is based on the parasite’s DNA (Caramori et al., 2022).

The low pathogenicity of *T. theileri* in healthy ruminants is generally attributed to its low parasitemia. However, immunocompromised, pregnant animals or those infected with bovine leukemia virus may exhibit high parasitemia, leading to clinical symptoms (Matsumoto et al., 2011). Cases of regenerative anemia, fever, and progressive weight loss in cattle positive for *T. theileri* have been reported in countries such as Spain, Ireland, Germany, and Italy (Hajihassani et al., 2020).

Therefore, the aim of the present study was to detect *Trypanosoma* spp. in naturally infected cattle from the municipality of Vassouras, Rio de Janeiro, Brazil, using blood smear and Woo’s technique, and to perform its molecular characterization.

## Material and Methods

Blood samples were collected from cattle in the municipality of Vassouras, Rio de Janeiro, Brazil (Latitude: 22° 24' 16'' S, Longitude: 43° 39' 48'' W). The minimum sample size was calculated based on the expected prevalence, using a sampling formula (Medronho et al., 2009). A 50% expected prevalence was assumed due to the absence of prior data, with a 95% confidence interval and a 7% margin of error, resulting in a minimum of 196 animals. The number of farms and animals sampled per farm was determined by convenience. Whole blood samples were collected from 226 dairy cattle of both sexes and different ages, from eight farms, via the coccygeal or mammary vein using the Vacutainer® system. The samples were stored in 4 mL tubes containing the anticoagulant ethylenediaminetetraacetic acid (EDTA). An aliquot of the EDTA-treated blood was used to determine the packed cell volume (PCV), according to the method described by Jain (1993), followed by application of Woo’s technique (Woo, 1970). Blood smears were also prepared, stained using a rapid staining method (Diff-Quick), and examined under an oil immersion objective (100x) for the detection of trypanosomatids. Another aliquot of whole blood was stored in 1.5 mL polypropylene microtubes and kept at -20°C until DNA extraction.

Total DNA was extracted from whole blood using the phenol-chloroform method described by Sambrook et al. (1989), with slight modifications. DNA samples were quantified using a NanoDrop 2000 spectrophotometer (Thermo Scientific^®^) and stored at -20°C for subsequent molecular analyses.

The 18S rRNA and 24Sα rRNA genes were used for the initial detection and taxonomic positioning of *Trypanosoma* spp., as these ribosomal markers are conserved and suitable for interspecific identification. In contrast, the cathepsin L-like (*CatL*) gene was selected due to its higher polymorphism and discriminatory power at the intraspecific level, allowing the genotyping of *T. theileri* isolates. The conventional PCR targeting a fragment of the 18S rRNA (Noyes et al., 1999) was performed in a final volume of 25 μL, using 1X Colorless GoTaq^®^ Buffer, 2 mM MgCl_2_, 0.2 mM of each dNTP, 0.4 μM of each primer, 1 U of GoTaq^®^ DNA Polymerase (Promega®), and 2 μL of DNA template. The thermocycling conditions were as follows: 94 °C for 5 minutes, followed by 35 cycles of 94 °C for 15 seconds, 55 °C for 30 seconds, and 72 °C for 60 seconds, with a final extension at 72 °C for 5 minutes. For molecular characterization, the 24Sα rRNA (Souto et al., 1999), PCR was performed in a final volume of 25 μL, using 1X Colorless GoTaq^®^ Buffer, 1 mM MgCl_2_, 0.2 mM of each dNTP, 0.2 μM of each primer, 1 U of GoTaq® DNA Polymerase (Promega®), and 2 μL of DNA template. The thermocycling conditions were as follows: 95 °C for 5 minutes, followed by 40 cycles of 94 °C for 30 seconds, 57 °C for 30 seconds, and 72 °C for 40 seconds, with a final extension at 72 °C for 5 minutes.

The *CatL* gene (Rodrigues et al., 2010; Yokoyama et al., 2015) was performed in a final volume of 25 μL, using 1X Colorless GoTaq® Buffer, 3 mM MgCl_2_, 0.2 mM of each dNTP, 0.4 μM of each primer, 1 U of GoTaq® DNA Polymerase (Promega®), and 2 μL of DNA template. The thermocycling conditions were as follows: 94 °C for 5 minutes, followed by 40 cycles of 94 °C for 30 seconds, 55 °C for 30 seconds, and 72 °C for 30 seconds, with a final extension at 72 °C for 5 minutes.

Positive controls for PCR reactions targeting the 18S rRNA and 24Sα rRNA genes consisted of *T. vivax*, and for *CatL*, *T. theileri*. These controls were obtained from naturally infected cattle confirmed by both blood smear and PCR and are part of the DNA bank of the Parasite–Host Interaction Laboratory, UFRRJ. The positive controls were sequenced to confirm their identity; however, these sequences were not deposited in public databases. Negative controls consisted of Nuclease-Free Water (Promega®).

Five microliters (5 µL) of PCR products treated with ExoSAP-IT (GE Healthcare®), following the manufacturer’s protocol, were sent for sequencing using the Sanger method. The fragments were sequenced in both directions using an automated genetic analyzer ABI 3500 Genetic Analyzer, Applied Biosystems^®^.

Chromatograms were analyzed, and nucleotide sequences were assembled and edited using CLC Main Workbench 23 software (CLC Bio-Qiagen, Aarhus, Denmark). The sequences were then submitted to identity analysis via the BLASTn program using the NCBI GenBank nucleotide database. Homologous sequences previously published in other studies were retrieved in FASTA format and aligned with the sequences obtained in this study using the ClustalW multiple alignment algorithm implemented in MEGA X software (Kumar et al., 2018). The aligned database was subsequently trimmed.

Phylogenetic analysis of *Trypanosoma* was performed based on datasets comprising 610 base pairs (bp) of the 18S rRNA gene with 24 Trypanosomatidae sequences; 257 bp of the 24Sα rRNA gene with 9 sequences; and 295 bp of the *CatL* gene with 23 sequences. The outgroup sequences used were *Bodo saltans* (MF962814) for 18S rRNA, *Leishmania donovani* (L19408) for the 24Sα rRNA gene, and *Leishmania infantum* (MW305434) for the *CatL* gene.

Phylogenetic trees were inferred using the Maximum Likelihood (ML) method, applying the Tamura-Nei substitution model with Gamma distribution (T93+G) for 18S rRNA; the Kimura 2-parameter model with Gamma distribution (K2+G) for 24Sα rRNA; and the Tamura 3-parameter model (T92) for the *CatL* gene. Analyses were performed in MEGA X (Kumar et al., 2018), with evolutionary model selection based on the Akaike Information Criterion (AIC). Statistical support for clades was estimated by bootstrap analysis with 1000 replicates using heuristic search. Final phylogenetic tree formatting was done using Inkscape v1.3.2.

## Results

A total of 226 blood samples were collected from dairy cattle of both sexes (Table 1), aged 45 days to 13 years, belonging to different breeds (*Bos taurus taurus*, Girolando (*Bos indicus x Bos taurus*), and crossbreds) from eight farms in Vassouras, Rio de Janeiro.

**Table 1:** Distribution of cattle sampled from each farm and sex (female/male).

**Farm**	**Total animals sampled**	**Female**	**Male**
**Farm 1**	19	10	9
**Farm 2**	21	12	9
**Farm 3**	30	30	0
**Farm 4**	60	60	0
**Farm 5**	10	6	4
**Farm 6**	12	9	3
**Farm 7**	42	38	4
**Farm 8**	32	29	3
**Total**	226	194	32

No trypomastigote forms were observed in blood smears, nor were any characteristic movements of flagellated organisms above the leukocyte layer in hematocrit capillaries, as described by Woo’s technique.

Molecular analyses using the 18S rRNA marker detected Trypanosomatidae in 0.88% (2/226) of the samples analyzed. The positive animals were crossbred females, aged 4 and 6 years, from the same farm, with packed cell volumes (PCV) of 36% (animal 36) and 27% (animal 37), within the species reference range (24–46%). The obtained 18S rRNA sequences (accession numbers PX352082–PX352083) showed 100% identity and 100% query coverage with *T. theileri* sequences from the United States (JX178185), Poland (KF765799), and Japan (LC522499).

For *T. theileri* characterization, sequences obtained for the 24Sα rDNA gene (accession number: PX353351-PX353352) exhibited 100% identity (100% query coverage) with a *T. theileri* isolate (XM_029023350). The sequence corresponding to the *CatL* gene (accession number: PX379569) showed 100% identity with sequences from Vietnam (AB742560; LC125447) and Sri Lanka (LC438508), clustering within genotype IB, lineage TthI.

**Figura 1:**
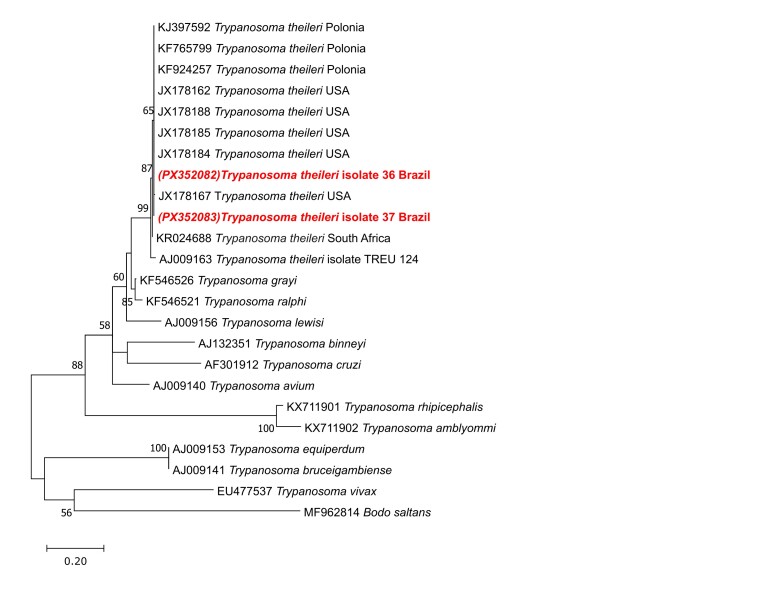
Phylogenetic tree based on a fragment of the 18S rRNA gene from cattle, Vassouras, RJ, Brazil, constructed using the Maximum Likelihood method and the Tamura–Nei model. Bootstrap values are shown next to the branches. Evolutionary rate differences among sites were modeled with a discrete Gamma distribution (+G, parameter = 0.3227). The scale bar indicates the number of substitutions per site. The analysis included 22 sequences and 646 aligned nucleotide positions.

**Figura 2:**
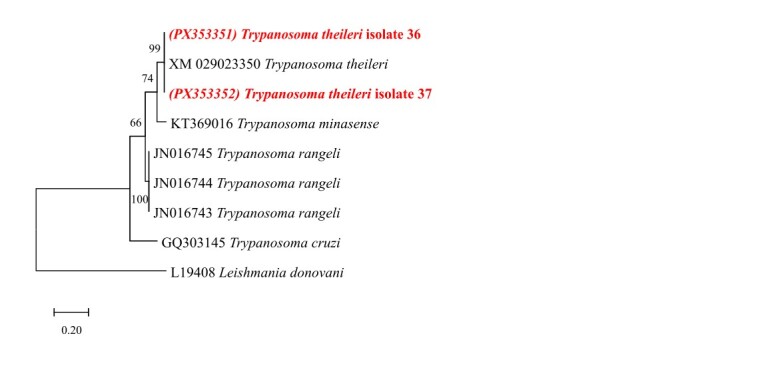
Phylogenetic tree based on a fragment of the 24Sα rRNA gene from cattle, Vassouras, RJ, Brazil, constructed using the Maximum Likelihood method and the Kimura 2-parameter model. Bootstrap values are shown next to the branches. Evolutionary rate differences among sites were modeled with a discrete Gamma distribution (+G, parameter = 1.7305). The scale bar indicates the number of substitutions per site. The analysis included 9 sequences and 257 aligned nucleotide positions.

**Figura 3:**
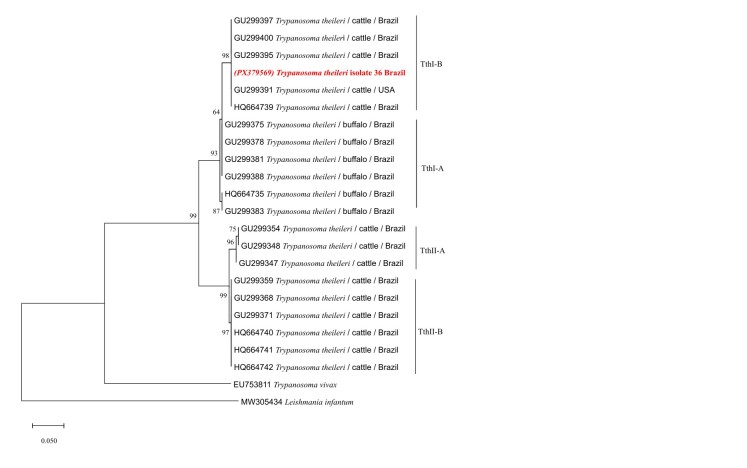
Phylogenetic tree based on a fragment of the *CatL* gene from cattle, Vassouras, RJ, Brazil, constructed using the Maximum Likelihood method and the Tamura 3-parameter model. Bootstrap values are shown next to the branches. The scale bar indicates the number of substitutions per site. The analysis included 23 sequences and 295 aligned nucleotide positions.

Phylogenetic analyses based on the 18S rRNA (610 bp) and 24Sα rRNA (257 bp) markers confirmed the identification of the isolates as *T. theileri*, supporting the existence of a widely distributed and genetically conserved lineage (Figures 1 and 2). For the 24Sα rRNA marker, only a limited number of sequences are available in GenBank for the amplified region, which restricted the number of sequences included in the phylogenetic analysis. Despite this genetic conservation, the phylogenetic positioning of the *CatL* (295 pb) gene within genotype IB (lineage TthI) reveals intraspecific variability, which may be relevant for understanding the host–parasite dynamics and the geographic structuring of *T. theileri* populations (Figure 3).

## Discussion

*Trypanosoma theileri* was first reported in the state of Rio de Janeiro in the Northwest Fluminense region (Santa Maria Madalena) by Gonçalves et al. (1998) and was later phylogenetically characterized by Abreu et al. (2024), who detected molecularly positive cattle in the South Fluminense (Barra do Piraí, Barra Mansa, and Valença), Northwest Fluminense (Santo Antônio de Pádua and Trajano de Moraes), and Serrana (Areal) regions. To date, no studies have reported the occurrence or molecular characterization of *T. theileri* in the municipality of Vassouras, South Fluminense region, Rio de Janeiro state. Nevertheless, given the geographic proximity among municipalities in this region and the frequent movement of cattle, the detection of *Trypanosoma* spp. in herds from Vassouras was expected, particularly because an outbreak of bovine trypanosomosis caused by *T. vivax* has previously been reported in this municipality (Costa et al., 2020).

In Brazil, studies on the prevalence of *T. theileri* in cattle are scarce. Previous investigations using parasitological diagnosis through hemoculture have reported the occurrence of the parasite in bovine populations (Pacheco et al., 2018). More recent studies employing molecular diagnostic techniques have also confirmed its presence in the state of Rio de Janeiro (Abreu et al., 2024). Most infections are subpatent and cannot be detected by blood smears or Woo’s technique, which detects only circulating parasitemia rather than latent infections, and this is consistent with the present study, in which no parasitic forms were observed using these methods. Therefore, negative parasitological results do not exclude the presence of infection. However, molecular techniques show higher sensitivity and specificity, allowing the detection of *T. theileri* even in cases of low parasitemia (De Gier et al., 2020). Nevertheless, although traditionally considered a parasite of low pathogenicity, *T. theileri* has been reported as an opportunistic agent in cases of co-infection (Pacheco et al., 2018), and increasing evidence indicates that it may contribute to disease under specific conditions such as stress, immunosuppression, or concomitant infections (Hajihassani et al., 2020). Despite the low prevalence observed, the detection of *T. theileri* in cattle from Vassouras is epidemiologically relevant, as it confirms the circulation of this parasite and expands the known geographic distribution of genotype TthIB within the state of Rio de Janeiro.

In Latin America, *T. evansi*, *T. vivax*, and *T. theileri* are the main *Trypanosoma* species affecting cattle (Rodrigues et al., 2006), highlighting the need to include *T. theileri* screening in cattle when infections by trypanosomatids are suspected. Care must be taken in the differential diagnosis of these species when based solely on the morphology of blood trypomastigotes, as mixed infections with *T. vivax*, *T. evansi*, and *T. theileri* may occur, and observers may lack adequate training to distinguish these forms in blood smears.

In South America, the genotype IA of *T. theileri* has been identified exclusively in buffaloes, while different genotypes have been described in cattle. In the Central and Southeast regions of Brazil, genotypes IC, IB, IIA, and IIB have been detected in cattle, whereas in the Northern region of the country and in Venezuela, only infections by genotype IIB have been observed in this host (Rodrigues et al., 2010). In the present study, detection of genotype IB corroborates findings by Abreu et al. (2024), who also identified this genotype in cattle from other municipalities in the state of Rio de Janeiro.

Despite the epidemiological relevance of these findings, some limitations of this study should be acknowledged. The sampling strategy was based on convenience, and no specific calculation was performed to determine the number of farms or animals per farm, which may limit the representativeness of the results. In addition, no endogenous gene was used as an internal control in the PCR assays, although DNA quality and concentration were assessed using a Nanodrop® spectrophotometer. These aspects should be considered when interpreting the results.

In conclusion, *T. theileri* is present in cattle from the municipality of Vassouras, Rio de Janeiro, with the identification of genotype IB classified within the TthI lineage. Notably, *T. vivax*, a highly pathogenic species associated with economically significant outbreaks, was not detected in any of the cattle samples analyzed.

## Data Availability

The entire dataset supporting the results of this study is available upon request to the corresponding author.
